# Effect of safranal on the response of cancer cells to topoisomerase I inhibitors: Does sequence matter?

**DOI:** 10.3389/fphar.2022.938471

**Published:** 2022-09-02

**Authors:** Lama Lozon, Ekram Saleh, Varsha Menon, Wafaa S. Ramadan, Amr Amin, Raafat El-Awady

**Affiliations:** ^1^ Sharjah Institute of Medical Research, University of Sharjah, Sharjah, United Arab Emirates; ^2^ College of Medicine, University of Sharjah, Sharjah, United Arab Emirates; ^3^ Clinical Biochemistry and Molecular Biology Unit, Cancer Biology Department, National Cancer Institute, Cairo University, Giza, Egypt; ^4^ Department of Biology, College of Science, UAE University, Al Ain, United Arab Emirates; ^5^ College of Pharmacy, University of Sharjah, Sharjah, United Arab Emirates

**Keywords:** safranal, topotecan, TDP1, topoisomerase, lung cancer and colon cancer

## Abstract

Lung and colorectal cancers are among the leading causes of death from cancer worldwide. Although topotecan (TPT), a topoisomerase1 inhibitor, is a first- and second-line drug for lung and colon cancers, the development of drug resistance and toxicity still remain as a major obstacle to chemotherapeutic success. Accumulating evidence indicates increased efficacy and reduced toxicity of chemotherapeutic agents upon combining them with natural products. We aimed to investigate the possible interaction of safranal (SAF), a natural compound obtained from *Crocus sativus* stigma, with TPT when used in different sequences in colon and lung cancer cell lines. The growth inhibitory effect of the proposed combination given in different sequences was assessed using the colony formation assay. The comet assay, cell cycle distribution, Annexin-V staining, and expression of proteins involved in DNA damage/repair were utilized to understand the mechanism underlying the effect of the combination. SAF enhanced the growth inhibitory effects of TPT particularly when it was added to the cells prior to TPT. This combination increased the double-strand break induction and dysregulated the DNA repair machinery, particularly the tyrosyl-DNA phosphodiesterase 1 enzyme. In addition, the SAF + TPT combination increased the fraction of cells arrested at the G2/M checkpoint as well as enhanced the induction of apoptosis. The current study highlights the status of SAF as a natural product sensitizing the lung and colon cancer cells to the cytotoxic effects of the anticancer drug TPT. In addition, it emphasizes the importance of sequence-dependent interaction which can affect the overall outcome.

## Introduction

Colorectal (CRC) and lung cancers are among the most prevalent cancer subtypes, together they account for approximately 2.8 million deaths from cancer annually (Sung et al., 2021). Chemotherapy is a principal option in the treatment of both types. However, its use is associated with various problems that limit its usefulness. First, they lack selectivity toward cancer cells, thus they can damage rapidly dividing normal cells including gastrointestinal tract epithelial cells and the bone marrow (Zugazagoitia et al., 2016; Falzone et al., 2018). Second, the development of resistance which can cause therapy failure (El-Awady et al., 2017; Mansoori et al., 2017). Combination treatment with different anticancer agents remains the core practice to overcome drawbacks of conventional cancer therapy. It allows the use of more than one agent in a reduced dosage which enhances the efficacy and reduces the likelihood of severe adverse events (Chabner and Longo, 2018). Recently, a new option of cancer therapy has emerged, which involves combining traditional chemotherapy with a naturally derived chemical that is showing evidence of cytotoxicity to cancer cells and limited damage toward normal cells (Huang et al., 2017, 2019; Rejhová et al., 2018). The sequence by which the combined agents are administered is as important as the choice of the agents themselves. This decision is based on understanding the pharmacodynamics and pharmacokinetics of the combined drugs (Mancini and Modlin, 2011; Poggio et al., 2017).

Topoisomerase I (TOPOI) inhibitors are an important class of chemotherapy, which include topotecan (TPT) and irinotecan. They are used in the management of different types of cancers such as colon, lung, and ovarian (Vennepureddy et al., 2015; Bailly, 2019). They exert their effect on TOPOI, which controls the topology of DNA and is usually required to relieve the DNA supercoiling to allow a flawless DNA replication and transcription. TOPOI works by generating a nick in a single strand of DNA double helix, rotating one strand over the other, and then it re-ligates the nick. TOPOI inhibitors stabilize the TOPOI–DNA cleavage complex (TOPcc) thus preventing DNA re-ligation, which results in a DNA single-strand break (SSBs). This will activate the SSB repair response that involves a cascade of proteins, among which is tyrosyl-DNA phosphodiesterase 1 (TDP1).

Human TDP1 is a member of the phospholipase D (PLD) superfamily and is described as a repair enzyme of TOPcc (Interthal et al., 2001). TDP1 repairs trapped TOPcc, caused by TOPOI inhibitors, by catalyzing the hydrolysis of 3̀̀-phosphotyrosyl bond located in that complex, to make DNA ends suitable for ligation (Pommier et al., 2014; Kawale and Povirk, 2018). Targeting TDP1 catches the attention of many scientists, suggesting that inhibiting TDP1 has the potential to augment the anticancer activity of TOPOI inhibitors by decreasing the repair of the stable complex caused by these drugs (Dean et al., 2014). This was supported by the finding that TDP1 knockout mice are hypersensitive to TOPOI inhibitors (Miao et al., 2006). It was reported that TDP1 tends to be overexpressed in non–small–cell lung cancer (NSCLC) and CRC, which confers resistance to TPT and other TOPOI inhibitors (Liu et al., 2007; Gilbert et al., 2012). The mutations in TDP1 that result in reduction of its catalytic activity sensitizes cancer cells to the cytotoxic effects of TOPOI inhibitors (Miao et al., 2006). These pieces of evidences suggest the importance of TDP1 in predicting the response to TOPOI inhibitors, and thus highlighting the significance of developing inhibitors to block its activity.

Safranal (SAF) constitutes the volatile fraction of *Crocus sativus* stigma (Leone et al., 2018). It has been shown to exert anticonvulsant, antidepressant, antihypertensive, antioxidant, and cytotoxic activities. These valuable effects illustrate its importance as a potential drug in future (Rezaee and Hosseinzadeh, 2013). In cancer, SAF shows a promising cytotoxic effect that is specific to cancer cells. This tumoricidal observation was seen even at low concentrations where it shows no toxicities (Riahi-Zanjani et al., 2015; Milajerdi et al., 2016). The mechanisms by which SAF is excreting its cytotoxicity on several cancer cell lines have been a hot area of research. In oral squamous cell carcinoma HSC-3 cells, SAF was able to reduce the invasiveness and migration of those cells by reducing the expression of mesenchymal markers and increasing those of epithelial cells (Zhang et al., 2017). In hepatocellular carcinoma cell HepG2, SAF shows ability to induce ER stress, to increase the extent of DNA double-strand breaks, and to increase apoptosis (Al-Hrout et al., 2018).

Inspired by the ability of SAF to potentially interfere with the function of TDP1 (Al-Hrout et al., 2018), we explored, in the present study, the effect of a new combination involving the TOPOI inhibitor TPT and SAF against HCT116 and A549 cell lines. The sequence-dependent effects of this combination and the mechanism of their cytotoxicity were also investigated.

## Materials and methods

### Cell lines and culture conditions

Two cancer cell lines (CRC: HCT116 and NSCLC: A549) were used in this study. The two cell lines were a generous gift from the Radiobiology and Experimental Radio-Oncology laboratory, University Cancer Center, Hamburg University, Hamburg, Germany. All cell lines were maintained in a RPMI (A549) or DMEM (HCT116) medium supplemented with 10% fetal bovine serum and 1% penicillin/streptomycin (Sigma-Aldrich, St. Louis, MO, United States) and are kept in a 37^ᵒ^C humidified incubator and an atmosphere of 5% CO_2_.

### Chemicals and antibodies

Topotecan hydrochloride (TPT) and safranal (SAF) (Sigma-Aldrich, Missouri, United States) were dissolved in dimethyl sulfoxide (DMSO) (Sigma-Aldrich, Missouri, United States), the stock solution of TPT was kept at −20°C. In preparation for an experiment, a serial dilution of TPT was prepared in the medium to achieve a concentration range of 0.001–1 μM and the concentration of DMSO in all samples was 0.02%. Primary monoclonal antibodies against γ-H2AX and TDP1 and secondary antibodies (anti-rabbit and anti-mouse) were obtained from Cell Signaling Technology (Danvers, MA, United States). In addition, propidium iodide (PI), RNAase, and crystal violet were purchased from (Sigma-Aldrich, Missouri, United States).

### Colony formation assay

SAF IC50 for each cell line was determined by CFA (El-Awady et al., 2010), and the cells were seeded in T25 cm^2^ culture flasks in densities ranging from 50 to 1,200 cells per flask. This was decided based on the SAF concentration used and the doubling time of the cells. After 24 h, the cells were treated with different SAF concentrations (10–200 μM) and the control group was treated with DMSO. All treatments were incubated for the whole period of colony formation. CFA was also used to assess the effect of the combination TPT/SAF in different sequences. The first sequence involves the addition of SAF IC50/IC25 24 h before TPT (0.001–1.0 μM) and the combination was incubated for 24 h. The second is where SAF IC50/IC25 is given simultaneously with TPT (0.001–1.0 μM) and the combination was incubated for 48 h. Last, SAF IC50/IC25 was introduced 24 h after TPT (0.001–1.0 μM) and incubated for another 24 h. In this experiment, the cells were seeded in T25 cm^2^ culture flasks with seeding densities ranging from 50 to 20,000 cells per flask. After 24 h, the cells were treated with SAF/TPT in different sequences. After 12–14 days when colonies of ≥50 cells were observed, the cells were fixed with 70% of ethanol for 30 min. After dryness, the flasks were stained with 1% crystal violet for 5 min at room temperature (RT), and then left to dry. The number of colonies at each treatment was counted using the microscope for the calculation of both plating efficiency (counted colonies/seeding number) and surviving fraction (plating efficiency of treated/plating efficiency of control). The IC50 values were calculated by sigmoidal curve fitting models using GraphPad Prism 3 software (GraphPad Software, San Diego, CA, United States). To assess the type of pharmacological interaction, the following isobologram equation was used (Berenbaum, 1989): I = d1/D1 + d2/D2, where I is the interaction index, d1 and d2 are the respective concentrations of SAF and TPT used in the combination required to produce 50% inhibition of cell growth. D1 and D2 are the concentrations of each agent alone that are able to yield the same degree of effect (50% inhibition of cell growth). If interaction index (I) < 1, the combination is synergistic, whereas if I = 1, it is additive and if I > 1, the effect is antagonistic.

### Neutral comet assay

To investigate the effect of SAF, TPT, and their combination on DNA damage, the neutral comet assay was performed according to the manufacturer’s protocol (Trevigen Inc, Gaithersburg, MD) following treatment with the most effective sequence, SAF IC25/IC50 added before TPT IC50. The cells were seeded in T25 cm^2^ flasks at a density of 0.5–0.8 million cells, after 24 h; they were treated with SAF IC25/IC50, and TPT was introduced 24 h thereafter. The combination was kept for 24 h, and then the cells were washed, harvested gently with a scraper, and counted to obtain 10^5^ cells/sample to be mixed with low melting point agarose (Trevigen Inc) at a proportion of 1:10. The cells/agarose mixture was evenly distributed on the comet slides and allowed to solidify before being immersed in lysis buffer (Trevigen Inc) at 4°C overnight. The next day, the slides were washed with the neutral buffer (Tris-base, sodium acetate, pH of 9), followed by 30 min/35 V electrophoresis at 4°C. The slides were submerged in a precipitation solution (7.5 M ammonium acetate) followed by 70% ethanol for 30 min each. The dry slides were then stained with SYBER Gold (Invitrogen, CA, United States) in TE buffer for 30 min, washed, dried, and covered. Images were captured at ×20 magnification using a confocal microscope (Olympus, Japan). The tail length and the intensity of the fluorescence signal in the tail area were measured using ImageJ (NIH, United States) for at least 70 cells/sample to calculate the tail moment (tail length + tail area).

### Western blot

The cells were seeded, treated as mentioned in the comet assay, and the total cell lysate was obtained by incubation with lysis buffer (Glycerol, 20% SDS and 1 M Tris, pH 6.8) containing the protease inhibitor cocktail (Sigma-Aldrich, United States). The protein was quantified using the DC™ protein assay kit (Bio-Rad, United States) and Western blot was performed as described previously (Ramadan et al., 2021). The samples containing equal amounts of protein (15–30 μg) were loaded into the gels to be separated on either 8% or 12% SDS polyacrylamide gel and transblotted onto the nitrocellulose membrane (Bio-Rad, United States). The membranes were blocked with 5% non-fat dried milk/1X TBS-Tween 20 and incubated with primary monoclonal antibodies (1:1,000) against γ-H2AX and TDP1 and β-actin overnight at 4°C, and then the membranes were blocked with 1X TBS-T. The secondary antibodies were prepared at a dilution of (1:2,000) and incubated with the membrane at RT for 1 h. Chemiluminescence was detected using the ECL method (Thermo Fisher Scientific, Massachusetts, United States) and developed using the ChemiDoc™ imaging system (Bio-Rad, United States). Quantification and analysis of the bands were performed using ImageLab™ software (Bio-Rad, United States).

### Cell cycle distribution analysis

The effect of SAF, TPT, and their combination on cell cycle progression was elucidated by flow cytometry (Saleh et al., 2009). The cells were seeded in T75 cm^2^ culture flasks at variable densities for each time point (0.5–1.5 million cells/flask). After 24 h, the cells were treated with DMSO, TPT IC50, SAF IC25/IC50, and a combination of them. The combinations were performed in three different sequences where SAF was given before TPT, concurrent or after TPT treatment for HCT116. However, for A549, only SAF before TPT was assessed. The flasks were incubated for different time intervals (12, 24, and 48 h). At each time interval, the cells were harvested and washed and fixed in 70% ethanol at 4°C. The fixed cells were washed two times with 1X PBS, counted and resuspended in 1X PBS containing RNAase (100 μg/ml), and incubated for 30 min at 37°C on a shaker. The cells were stained with propidium iodide (PI) (50 μg/ml) and analyzed using an Accuri C6 flow cytometer (Becton Dickenson, United States). DNA histograms were obtained using FlowJo V.10 software (Tree Star, Inc-Oregon, United States).

### Apoptosis assay

The induction of apoptosis in HCT116 and A549 cells after treatment with DMSO, TPT IC50, and SAF IC25/IC50 alone or in combination (SAF plus TPT) was assessed for the percentage of cells positive for either Annexin-V, PI or both. The cells were prepared according to the manufacturer’s protocol FITC Annexin-V Apoptosis Detection Kit (BD Biosciences, United States). A measure of 5 µl of FITC Annexin-V and 10 µl PI (500 mg/ml) was added and the samples were incubated for 15 min in the dark at RT. Binding buffer (400 µl) was then added and cell staining was analyzed using an Accuri C6 flow cytometer (Becton Dickenson, United States).

### Statistical analysis

All experiments were carried out in triplicate and repeated at least three times. Data are expressed as means ± SEM. Statistical analysis was performed by unpaired student’s *t*-test using GraphPad Prism 3 (CA, United States) software (GraphPad Software). *p* < 0.05 was considered statistically significant.

## Results

### Effect of SAF on the survival of HCT116 and A549 cells

**FIGURE 1:**
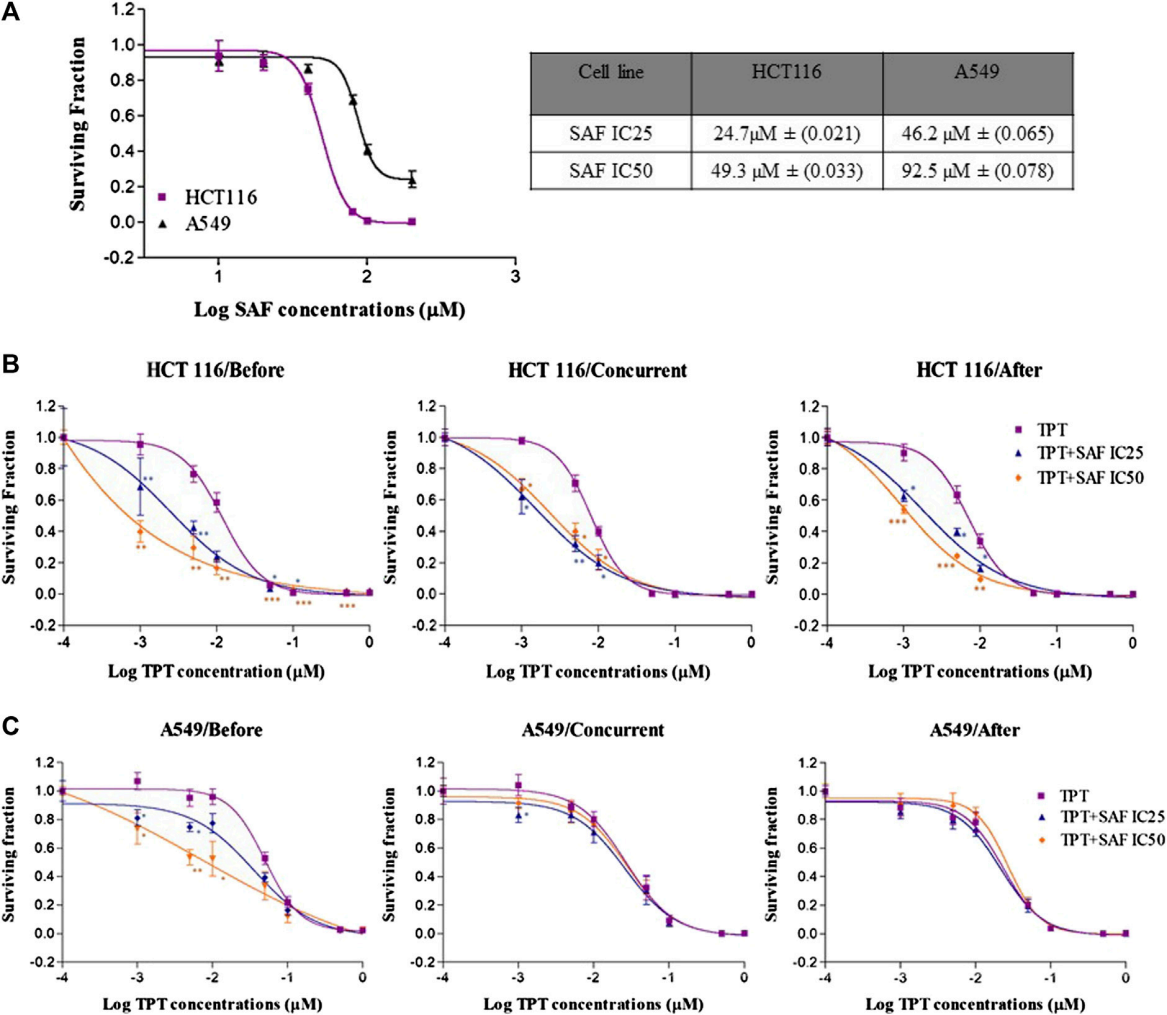
Effect of SAF IC25/IC50 on the sensitivity of both HCT116 and A549 cells to topotecan treatment by using different sequences. **(A)** Colony formation assay was used to measure the sensitivity of HCT116 and A549 cancer cell lines to different concentrations of SAF (µM). **(B–C)** HCT116 and A549 cells were treated with IC25/IC50 concentrations of SAF either 24 h before, concurrently or 24 h after treatment with different concentrations of TPT (µM). Surviving fraction is calculated by dividing the plating efficiency (PE) of treated cells by the (PE) of control cells. The means ± SEM of at least two independent experiments are shown. *p < 0.05, **p < 0.005, and ***p < 0.0005 vs TPT group.

The growth inhibition of HCT116 and A549 cells by SAF treatment was assessed using the colony formation assay. SAF treatment caused concentration-dependent reduction in survival of both cell lines (Figure 1A). The IC50 of SAF was determined for each cell line using the best fitting curve method in prism software. Based on the calculated IC50 value, HCT116 is more sensitive to SAF with IC50 of 49.3 µM while A549 is more resistant with IC50 of 92.5 µM.

### Effect of SAF on the cytotoxic effect of TPT on HCT116 and A549 cells

The effect of SAF and TPT combination treatment given in three different sequences on the survival of the HCT116 and A549 cells was evaluated using the colony formation assay, where the IC25 and IC50 of SAF equivalent to (24.7/49.3 µM) for HCT116 and to (46.3/92.5 µM) for A549 cells were used in combination with different concentrations (0.0–1.0 µM) of TPT. This treatment scheme was applied for three different sequences: SAF 24 h before TPT, SAF, and TPT were added simultaneously and SAF 24 h after TPT. IC50 of TPT was 0.01 µM for HCT116 cells and 0.05 µM for A549 cells. This indicates that A549 cells are more resistant to TPT compared to HCT116 cells. The combined treatment of SAF and TPT was able to decrease the IC50 of TPT in all treatment sequences in HCT116 cells (Figure 1B and Table 1) while in A549, only when SAF was given before TPT there was a significant reduction in the IC50 of TPT (Figure 1C and Table 1). It is worth mentioning that incubation of HCT116 cells with SAF 24 h before the addition of TPT resulted in 16.8 fold increase in the sensitivity of the cells to TPT (IC50 of TPT alone is 0.01 µM compared to 0.00069 µM for SAF + TPT) (Figure 1B) (Table 1). The type of pharmacological interaction between SAF and TPT was determined using the isobologram equation. In HCT116 cells, the interaction between SAF IC25 and TPT in all treatment sequences was synergistic, while the interaction of SAF IC50 with TPT was additive when SAF was given before TPT. For A549 cells, the interaction of SAF IC25/IC50 was additive when SAF was added before TPT, while the other sequences showed an antagonistic interaction (Table 2).

**TABLE 1:** IC50 values after treatment of HCT116 and A549 cell lines with TPT and TPT + SAF at different sequences. IC25 (μM): the concentration of the drug necessary to produce 25% inhibition of cell growth. IC50 (μM): the concentration of the drug necessary to produce 50% inhibition of cell growth. Represented data are means ± SEM of at least three independent experiments.

	IC50 (µM)	
Cell line	HCT116	A549
TPT alone	0.0100 ± (0.0013)	0.0496 ± (0.0067)
SAF IC25+TPT		
Before	0.0028 ± (0.0004)	0.0280 ± (0.0077)
Concurrent	0.0018 ± (0.0010)	0.0226 ± (0.0194)
After	0.0021 ± (0.0002)	0.0191 ± (0.0033)
SAF IC50+TPT		
Before	0.0007 ± (0.0006)	0.0098 ± (0.0184)
Concurrent	0.0025 ± (0.0017)	0.0257 ± (0.0103)
After	0.0012 ± (0.0002)	0.0258 ± (0.0009)

**TABLE 2:** Effect of SAF on TPT cytotoxicity in HCT116 and A549 cells and the type of interaction after each treatment and sequence. Interaction index: *I* = d1/D1 + d2/D2, where d1 and d2 are the respective concentrations of TPT and SAF used in the combination required to produce a fixed level of inhibition IC25/IC50. While D1 and D2 represent concentrations of each TPT and SAF alone to produce the same magnitude of effect (IC25/IC50). Values presented are means ± SEM of at least three independent experiments.

IC50 (µM)				
Cell line	HCT116		A549	
SAF IC25+TPT	Interaction index (I)	Type of interaction	Interaction index (I)	Type of interaction
Before	0.76 ± (0.03)	Synergy	1.04 ± (0.069)	Additivity
Concurrent	0.73 ± (0.04)	Synergy	1.33 ± (0.068)	Antagonistic
After	0.80 ± (0.1)	Synergy	1.40 ± (0.058)	Antagonistic
SAF IC50+TPT				
Before	1.00 ± (0.038)	Additivity	1.05 ± (0.013)	Additivity
Concurrent	1.32 ± (0.07)	Antagonistic	1.95 ± (0.085)	Antagonistic
After	1.17 ± (0.1)	Antagonistic	2.21 ± (0.102)	Antagonistic

### Effect of SAF, TPT, and their combination on the induction of DNA damage

**FIGURE 2:**
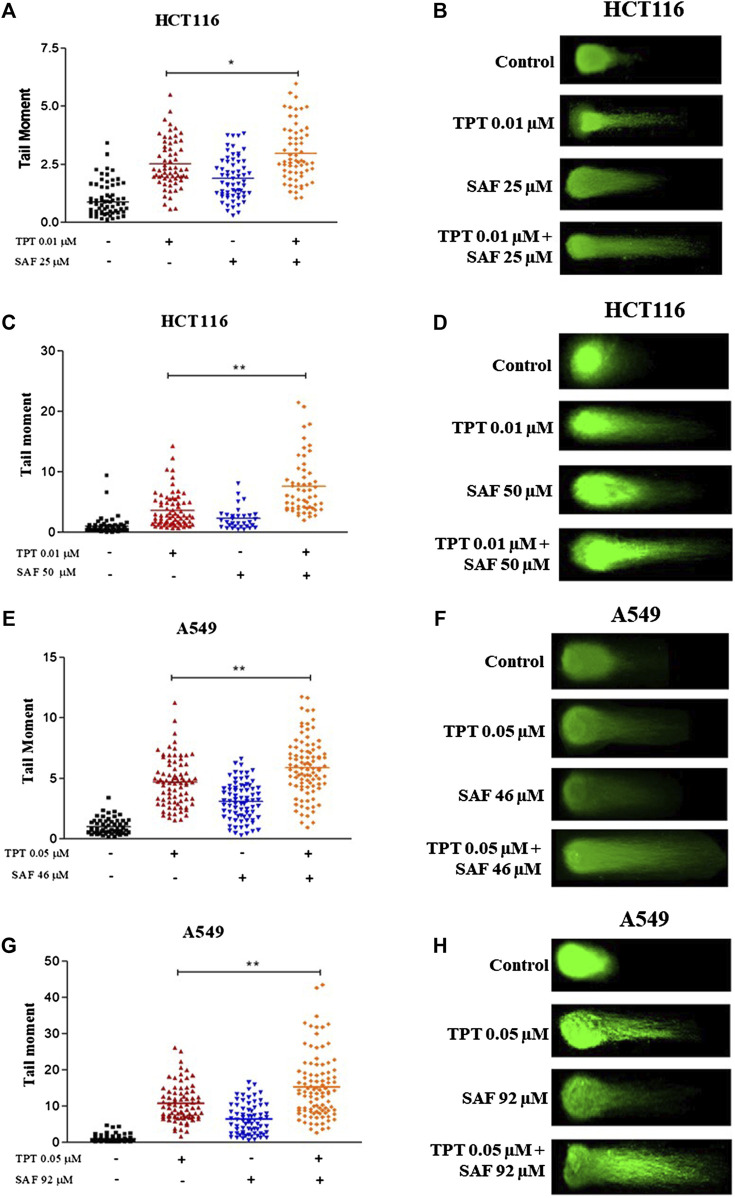
Neutral comet assay of HCT116 and A549 cells treated with SAF IC25/IC50 before TPT IC50 treatment for 24 h. **(A–D)** Tail moment and fluorescence microscopic images of HCT116 cells treated with SAF IC25/IC50, 24 h before TPT 0.01 µM, the combined treatment was incubated for 24 h. **(E–H)** Tail moment and florescence microscopic images of A549 cells treated with SAF IC25/IC50, 24 h before TPT 0.05 µM and the combined treatment was incubated for 24 h. The means ± SEM of at least two independent experiments are shown. *p < 0.05, **p < 0.005 vs TOP group.

**FIGURE 3:**
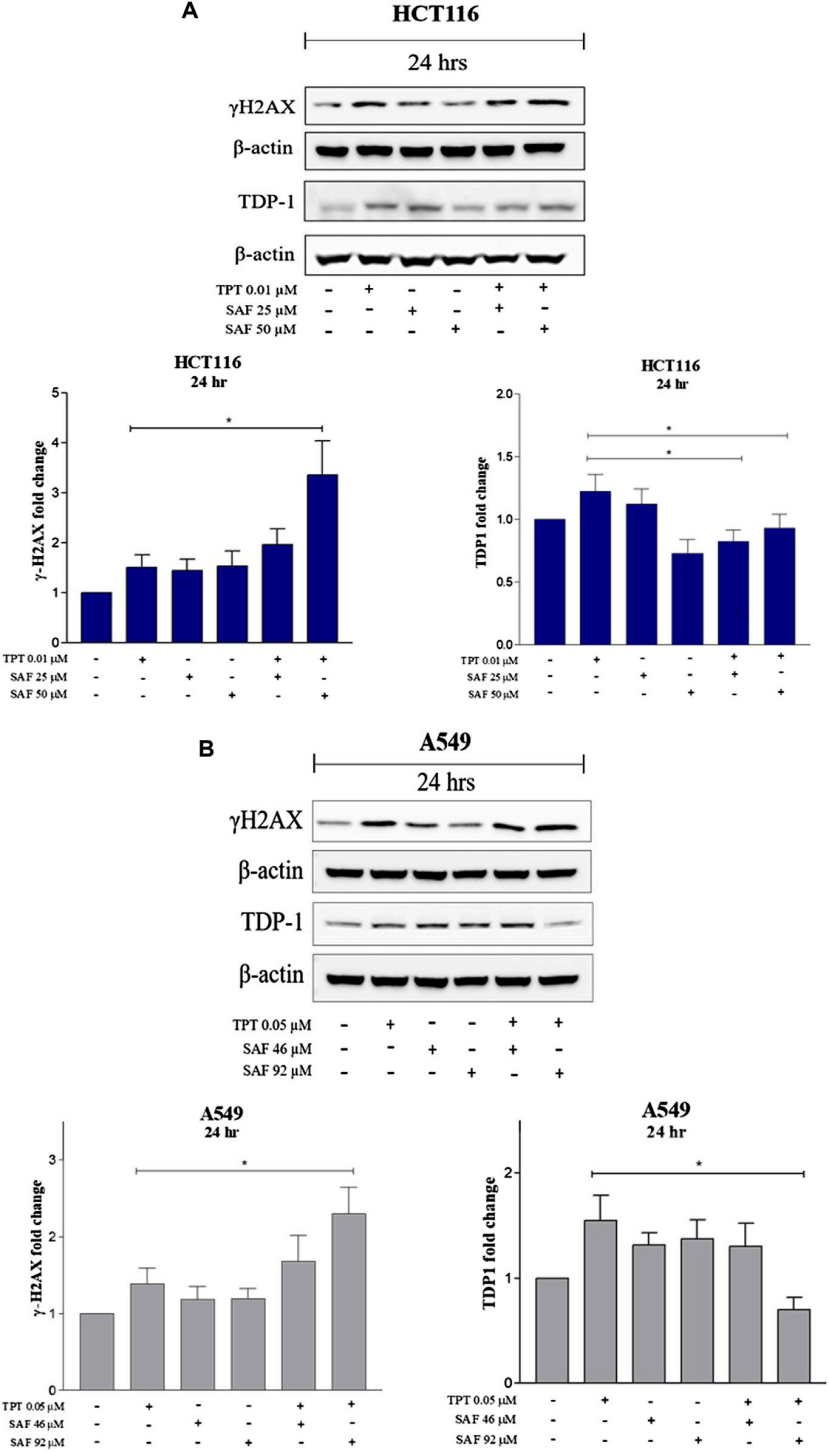
Expression of γH2AX and TDP1 in HCT116 and A549 cells after treatment with SAF IC25/IC50 before TPT IC50 treatment Western blot analysis for HCT116 **(A)** and A549 **(B)** was performed by loading the whole cell lysates treated with SAF IC25/IC50 before TPT 0.01 µM for 24 and 48 h in HCT116 cells and treated with SAF IC25/IC50 before TPT 0.05 µM for 24 and 48 h in A549 cells. Bar graphs represent densitometric quantifications of Western blot bands normalized to β-actin and vehicle-treated control. The means ± SEM of at least three independent experiments are shown. *p < 0.05 vs. TPT group.

TPT exerts its cytotoxic effect via induction of single-strand breaks (SSBs) which are converted to double-strand breaks (DSBs) during DNA replication. So, we next investigated the contribution of SAF to the overall seen DNA damage upon combination given in the most effective sequence using the neutral comet assay. Both HCT116 and A549 cells were treated with SAF IC25/IC50 24 h before the addition of TPT IC50, then the combined therapy was incubated for another 24 h. HCT116 cells showed a significant increase in the extent of DNA damage (DSBs) in the combined treatment compared to TPT 0.01 µM alone with both SAF IC25/IC50 (Figures 2A–D). However, the effect was more pronounced with SAF IC50 combination (Figure 2C). In A549 cells, both SAF IC25/IC50 combined with TPT 0.05 µM caused a similar increase in DSBs induced compared to TPT alone (Figures 2E–H). The phosphorylated H2AX histone protein (γH2AX) normally forms nuclear foci at DNA break sites in cells experiencing DNA damage. It is, therefore, used as a DNA damage marker and was used in the current study to confirm the finding from the comet assay. HCT116 cells were treated with SAF IC25/IC50 given 24 h before TPT 0.01 µM. There was a significant increase in the level of γH2AX after the combined treatment with SAF IC50 and TPT 0.01 µM (Figure 3A). A similar pattern of increase in γH2AX was also seen in A549 cells (Figure 3B).

### Effect of the combined treatment of SAF and TPT on the modulation of DNA repair

TDP1 is a key enzyme required to repair SSBs induced by TPT. SAF was reported to bind to TDP1 in an inhibitory manner (Al-Hrout et al., 2018). Thus, we investigated the changes in the expression level of TDP1 following treatment with SAF IC25/IC50 given before TPT IC50. In both cell lines (HCT116 and A549), the combined treatment of SAF IC50 and TPT IC50 significantly reduced the expression of TDP1 (Figures 3A,B). However, the combination IC25 SAF with TPT reduced the expression of TDP1 in HCT116 only (Figure 3A).

### Effect of the combined treatment of SAF plus TPT on the cell cycle progression

**FIGURE 4:**
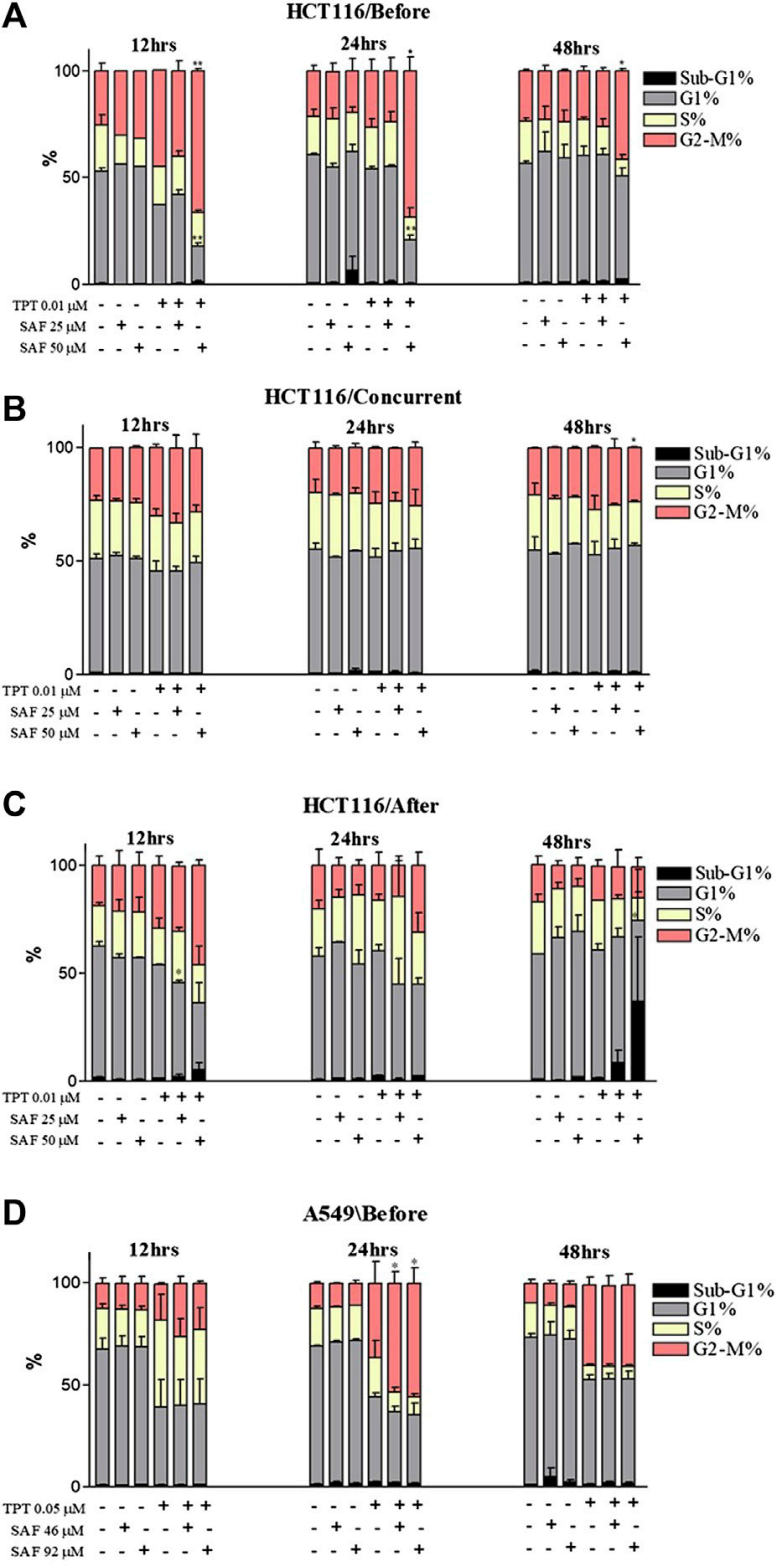
Cell cycle distribution analysis of HCT116 and A549 cells treated with SAF IC25/IC50 and TPT IC50 µM for different time intervals and sequences. **(A–C)** HCT116 cells treated with SAF IC25/IC50 24 h before TPT 0.01 µM **(A)**, concurrently with TPT 0.01 µM **(B)**, and 24 h after TPT 0.01 µM **(C)**, the combined treatment was incubated for 12, 24, and 48 h. **(D)** A549 cells treated with SAF IC25/IC50 24 h before TPT 0.05 µM, the combined treatment was incubated for different time intervals of 12, 24, and 48 h. The means ± SEM of at least two independent experiments are shown. *p < 0.05, **p < 0.005 vs TPT group.

Cell cycle distribution analyses of both cell lines at 12, 24, and 48 h intervals were performed to determine the effect of combining SAF IC25/IC50 with TPT at the sequences that showed a significant reduction in the IC50 of TPT in the proliferative assay. The combination of SAF IC50 and TPT 0.01 µM in HCT116 cells caused an increase in the fraction of cells arrested at the G2/M checkpoint at 12, 24, and 48 h when SAF was given before TPT (Figure 4A and Supplementary Figure S1A). The same effect was achieved at 48 h only in the treatment sequence where SAF is given simultaneously with TPT (Figure 4B and Supplementary Figure S1B). SAF IC25 was only able to increase in the S phase arrest when given after TPT 0.01 μM at the 12 h interval (Figure 4C and Supplementary Figure S1C). For A549 cells, treatment with SAF IC25/IC50 before 0.05 µM of TPT showed a statistically significant increase in the fraction of cells arrested at the G2/M checkpoint at 24 h post-treatment compared to TPT 0.05 µM alone only (Figure 4D and Supplementary Figure S1D). Other time points did not show any change in the cell cycle distribution among single or combination treatments (Figure 4D).

### SAF enhances induction of apoptosis when combined with TPT

**FIGURE 5:**
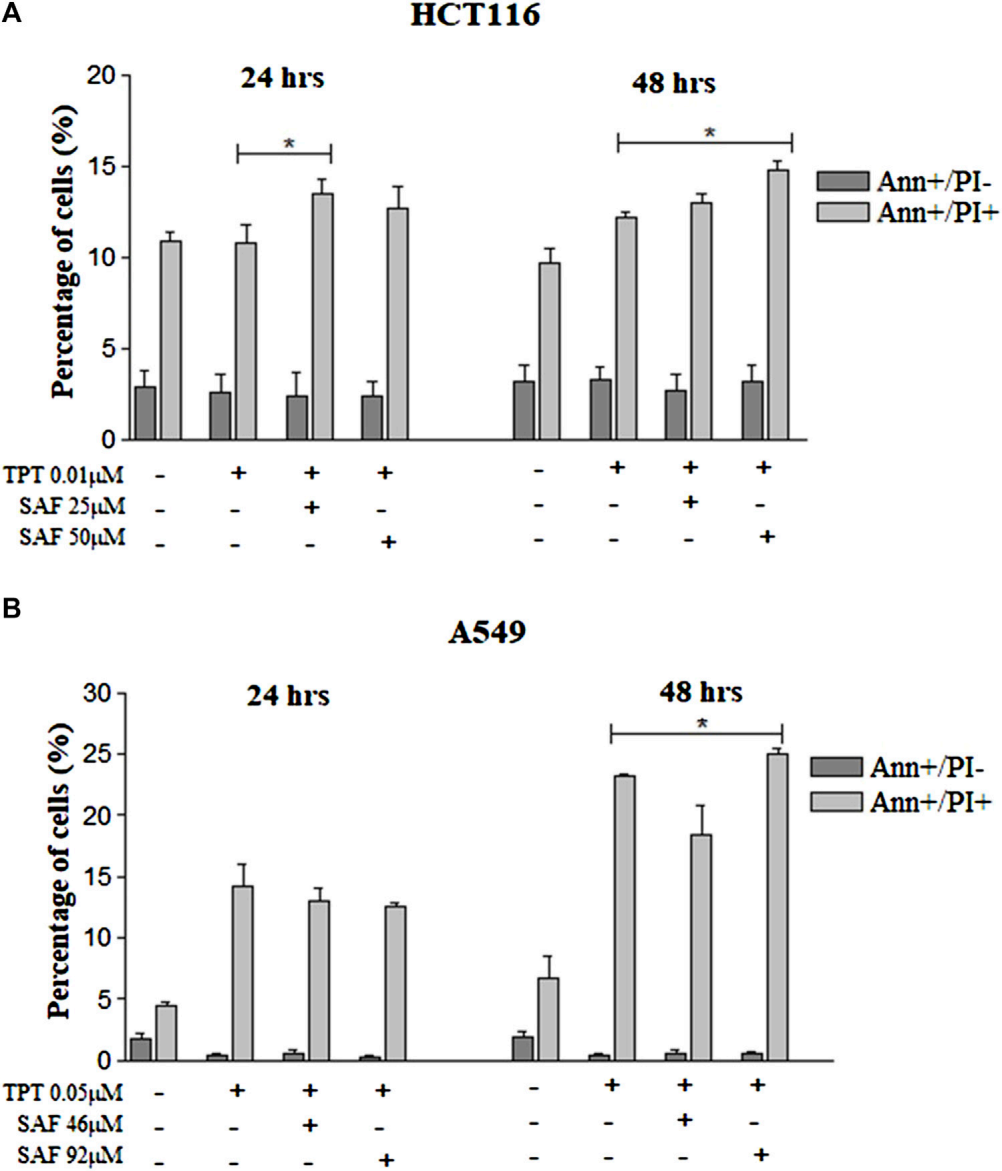
Annexin-V/PI flow cytometry of HCT116 and A549 cells treated with SAF IC25/IC50 before TPT IC50 treatment. Annexin-V/PI analysis of apoptosis in HCT116 cells **(A)** treated with SAF IC25/IC50 24 h before TPT 0.01 µM treatment. The combined treatment was incubated for 24 and 48 h. The cells were stained with fluorescein-conjugated Annexin-V and propidium iodide (PI) and analyzed by flow cytometry. The same analysis was performed for A549 cells **(B)** treated with SAF IC25/IC50 24 h before TPT 0.05 µM treatment. The means ± SEM of at least three independent experiments are shown. *p < 0.05 vs. TPT group.

The main proposed mechanism of safranal’s cytotoxicity is its ability to induce apoptosis. So, we performed Annexin-V staining for HCT116 cells which revealed an increase in Annexin+/PI + cells at 24 and 48 h with SAF IC25/IC50 combined with TPT which indicates late apoptosis (Figure 5A and Supplementary Figure S2A). The same results were observed when A549 cells were treated with SAF IC50 given before TPT at 48 h (Figure 5B and Supplementary Figure S2A).

## Discussion

Resistance of cancer cells and the low safety profile of anticancer drugs are two major factors limiting the success of cancer therapy. Combining natural products with current anticancer drugs is one strategy to overcome this problem. Due to their high safety profile, natural products will not add to the toxicity of anticancer drugs, yet they might enhance their anticancer activity by allowing the use of smaller doses and enhancing the therapeutic index of anticancer drugs. The clinical applications of TOPO1 inhibitors are limited to confined tumor subtypes due to profound neutropenia and bone marrow suppression associated with the effective dose (Chabner and Longo, 2018). Recent data have shown that high levels/activity of TDP1 can negatively impact the success of therapy with TOPO1 inhibitors as there is a higher degree of TOPO1-damage reversal thus negating the ensuing DNA damages and cell death signal imparted in the cancer cell. TOPO1 vs. TDP1 ratio has recently become an important indicator/predictor of response to TOPO1 inhibitors (Meisenberg et al., 2014). High TOPO1 levels with low TDP1 levels/activity are an ideal scenario for the enhanced use of TOPO1 inhibitors as an effective anticancer strategy. Identifying TDP1 inhibitors to reduce the denominator in this ratio would extend the therapeutic benefit of the TOPO1 inhibitors. A recent study has revealed that using an *in silico* molecular docking can help SAF bind to the active site of TDP1 suggesting its ability to prevent TDP1 to correct single-strand breaks induced in the DNA. The same study showed that SAF can reduce the expression levels of TDP1 in HepG2 cells (Al-Hrout et al., 2018). In the present study, we tested the effect of the natural product SAF on the anticancer activity of the TOPOI inhibitor TPT. Moreover, the effect of combining SAF with TPT in different sequences was investigated.

In the current study, incubation of the colon cancer cell line (HCT116) or the non–small–cell lung cancer cell line (A549) with SAF (IC25 or IC50) 24 h prior to TPT increased the amount of DNA double-strand breaks compared to cells treated with TPT alone as indicated by the increased tail moment (Figure 2) and the increased γ–H2AX formation (Figure 3). This may be attributed to additional induction of DNA DSB by SAF or due to the ability of SAF to inhibit the repair of TPT-induced DSBs or both.

Our results show that treatment of both cell lines with TPT alone increases the expression of TDP1, whereas combination of SAF + TPT (SAF IC25/IC50 followed by TPT IC50) reduced the expression of TDP1. This may explain the increased sensitivity of both cell lines to TPT when cells were treated with SAF followed by TPT. The reduced expression of TDP1 in combined treatment decreases the repair of TPT-induced DNA lesions and results in accumulation of more DNA lesions leading to enhanced cell death.

It is noteworthy that the sequence of adding SAF and TPT to the cells significantly affected the type of pharmacological interaction between the two compounds. Incubating the cells with SAF before TPT showed the best interaction (synergistic or additive), whereas other sequences (concurrent treatment or TPT followed by SAF) resulted in antagonistic interaction in most cases. The sequence-dependent type of pharmacological interaction of anticancer drugs with other compounds has been previously reported (El-Awady et al., 2011).

The inability of cancer cells to efficiently repair drug-induced DNA damage stimulates cell cycle checkpoints. The main aim was to prevent cells from entering the S and M phases of the cell cycle with damaged DNA (El-Awady et al., 2011). In the present investigation, incubation of cells with SAF, especially before TPT resulted in an increased fraction of cells arrested at the G2 phase of the cell cycle compared to cells treated with TPT alone. TOPOI inhibitors are known to be most toxic to actively dividing cells and to induce lethal DNA lesions during the S phase of the cell cycle (Pommier, 2006). This explains the high fraction of cells arrested at the G2 phase upon treatment with SAF + TPT. Inhibition of TDP1 by SAF reduces the repair of TPT-induced DNA damage and activates the G2/M checkpoint to prevent cells from entering the M phase with damaged DNA. The cells arrested at the G2 phase are given more time to repair their DNA lesions or to be removed by different cell death pathways such as apoptosis. Combined treatment with SAF followed by TPT increased the fraction of apoptotic and necrotic cells in both cell lines indicating the stimulation of apoptosis/necrosis pathways upon combining SAF with TPT. This is in line with previous studies showing activation of apoptosis upon treatment of cells with topoisomerase inhibitors (El-Awady et al., 2008).

In conclusion, our results emphasize the importance of SAF as a candidate-sensitizing agent of colon and lung cancer cells to the effect of topotecan. This sensitization showed to be sequence-dependent with the most profound effects when SAF is given before TPT. Furthermore, it gives an insight into understanding the mechanism of the potentiated growth inhibitory effects seen with combination treatment that involves DNA damage, DNA repair machinery, cell cycle, and apoptosis. Patients with colon or lung cancer can greatly benefit from the results of this study by combining SAF with TOPOI inhibitors to enhance the anticancer effect and to improve the safety profile of the TOPOI inhibitors.

## Data Availability

The original contributions presented in the study are included in the article/Supplementary Material; further inquiries can be directed to the corresponding author.
