# Supplementary figures and images for: Effect of safranal on the response of cancer cells to topoisomerase I inhibitors: Does sequence matter?
Source: Front Pharmacol. 2022 Sep 2;13:938471. doi: 10.3389/fphar.2022.938471 (PMC9479137; doi:10.3389/fphar.2022.938471)

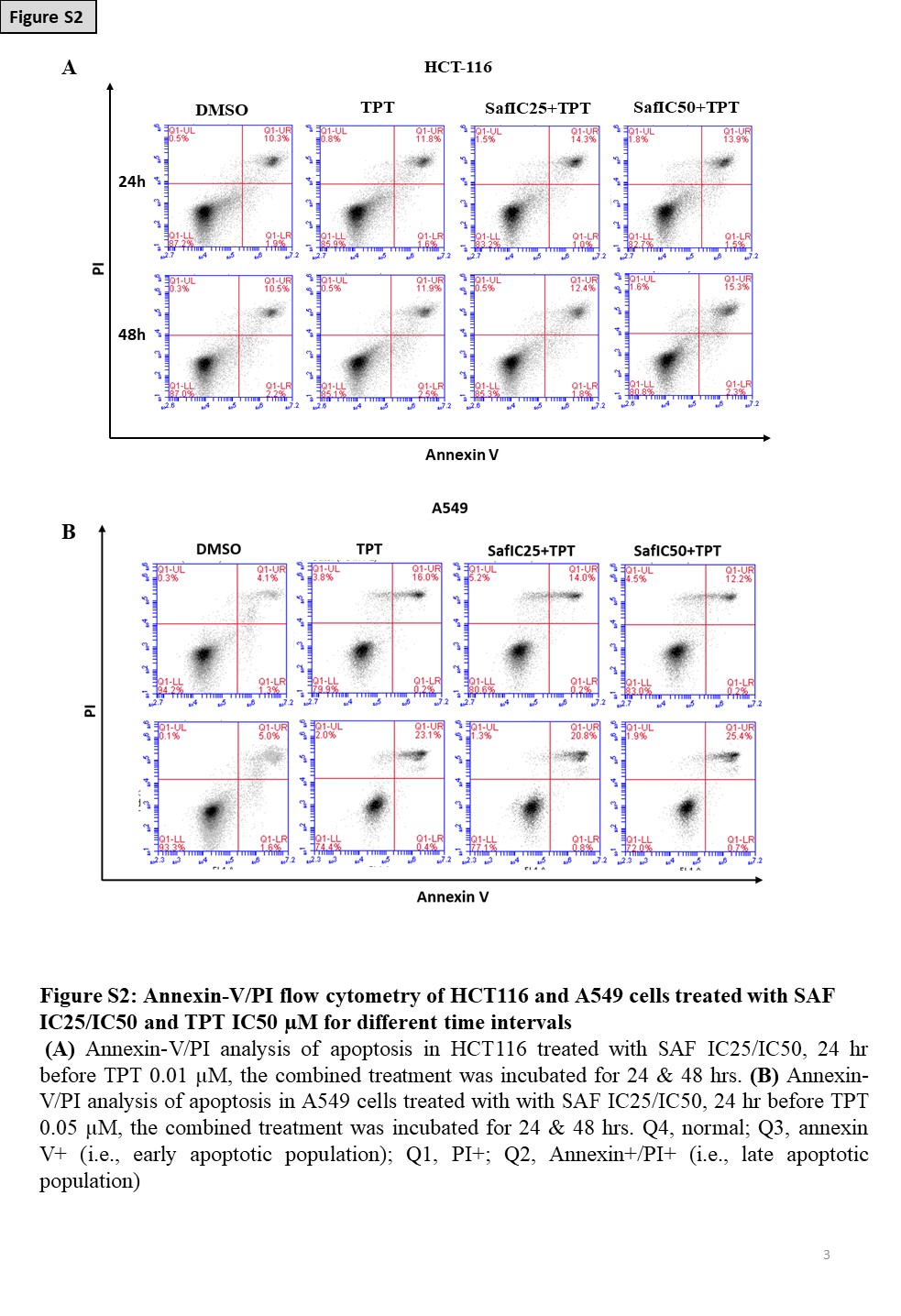

Supplement: Supplementary file 1 [file Image3.JPEG]

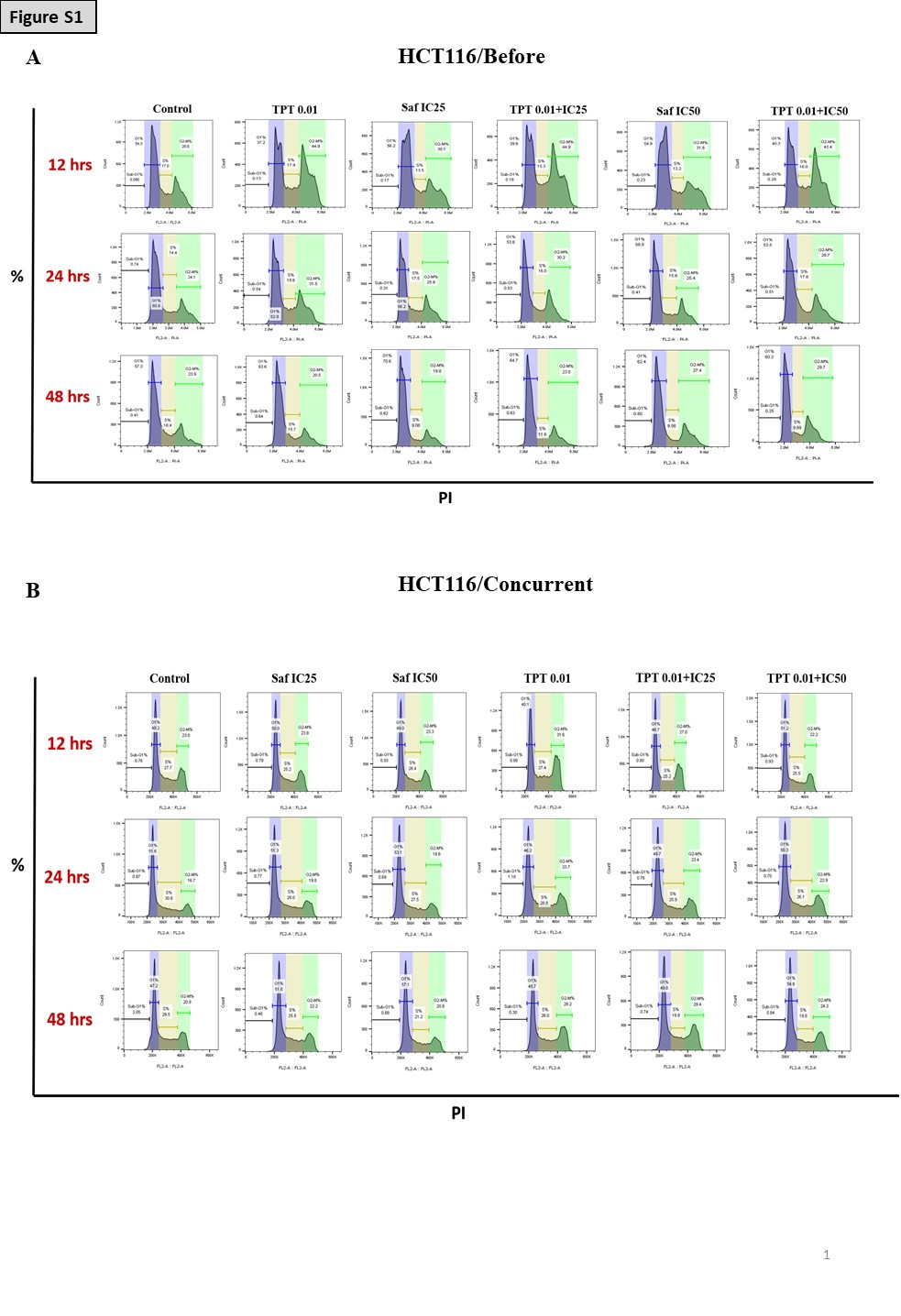

Supplement: Supplementary file 2 [file Image1.JPEG]

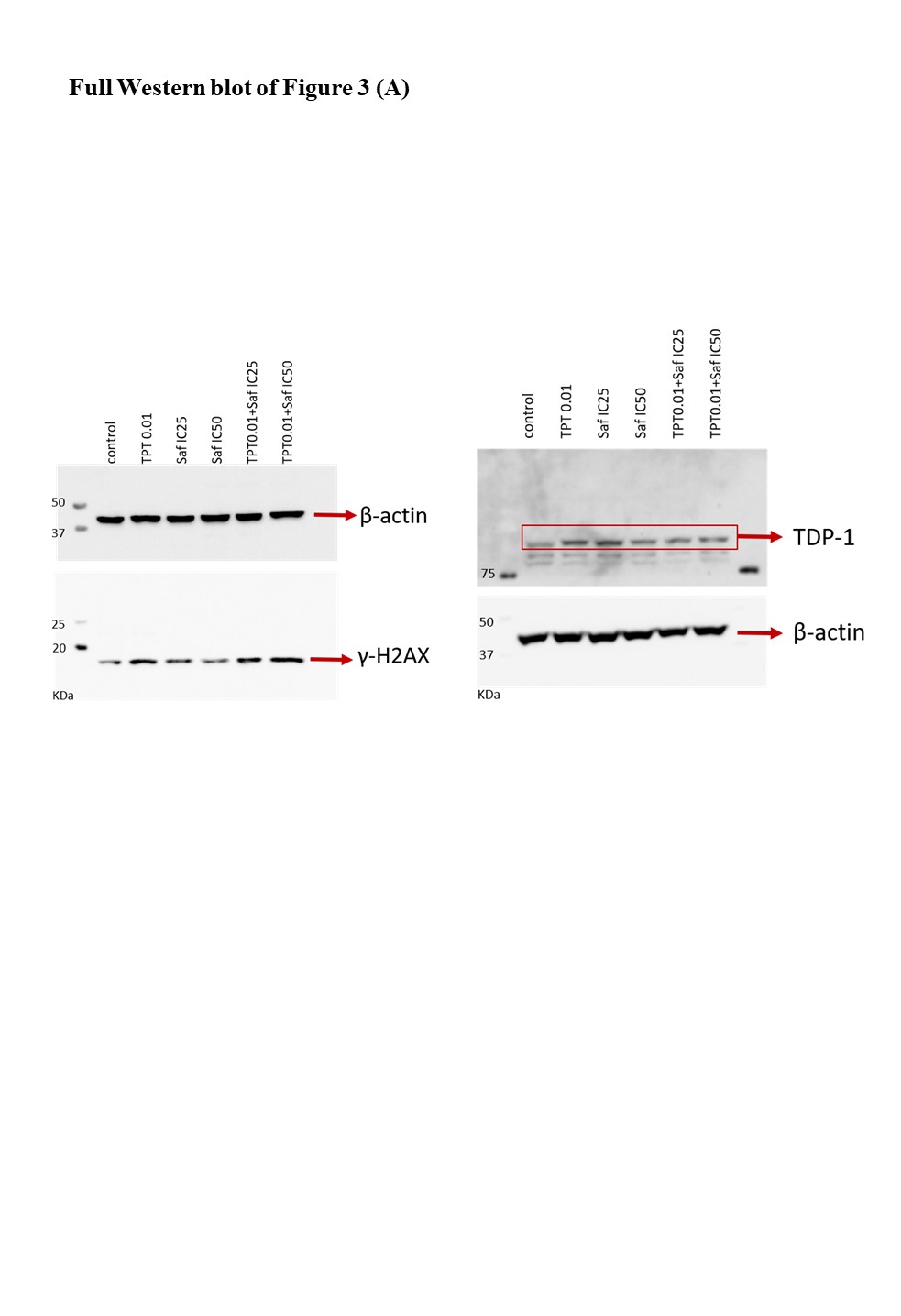

Supplement: Supplementary file 3 [file Image4.JPEG]

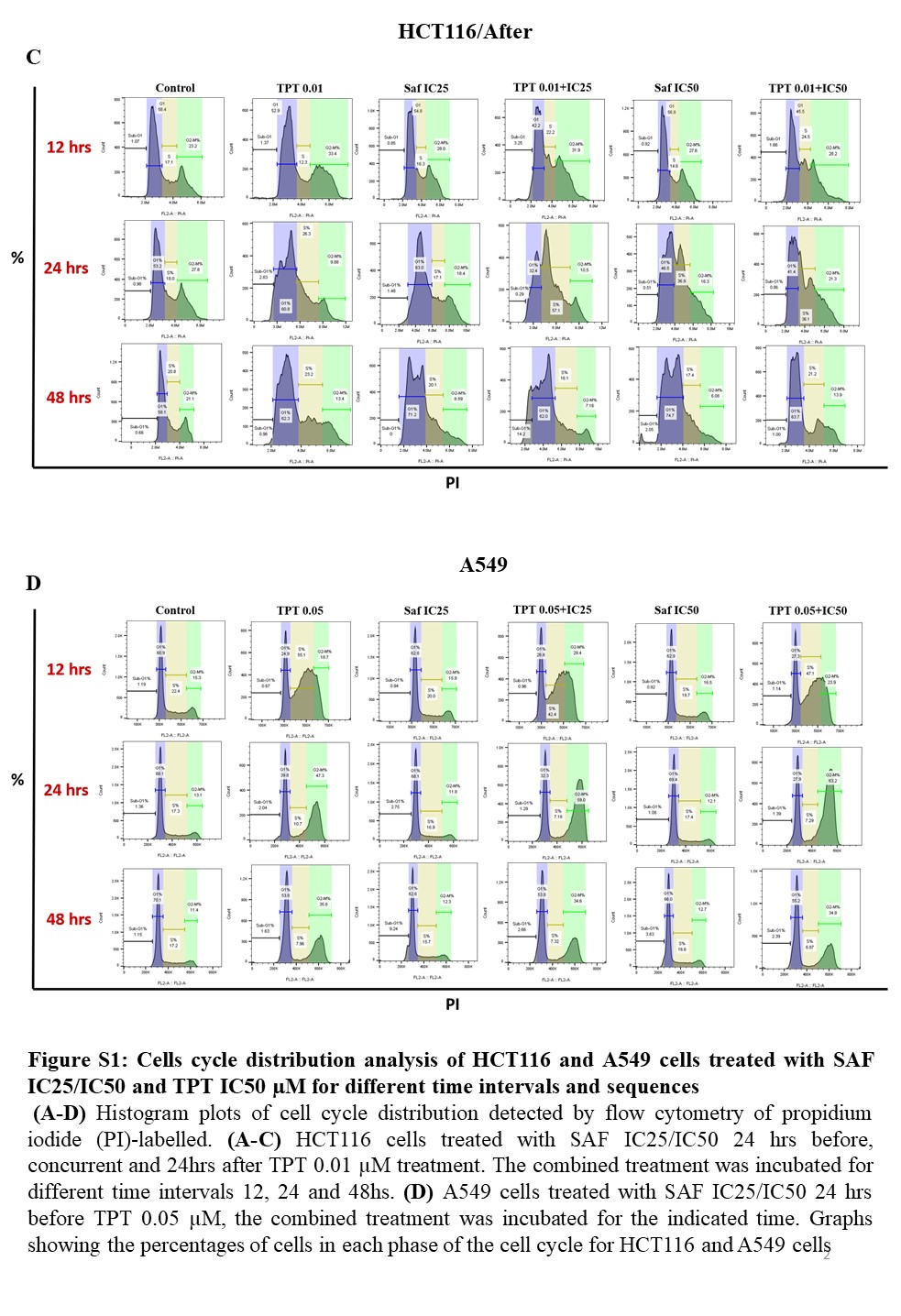

Supplement: Supplementary file 4 [file Image2.JPEG]

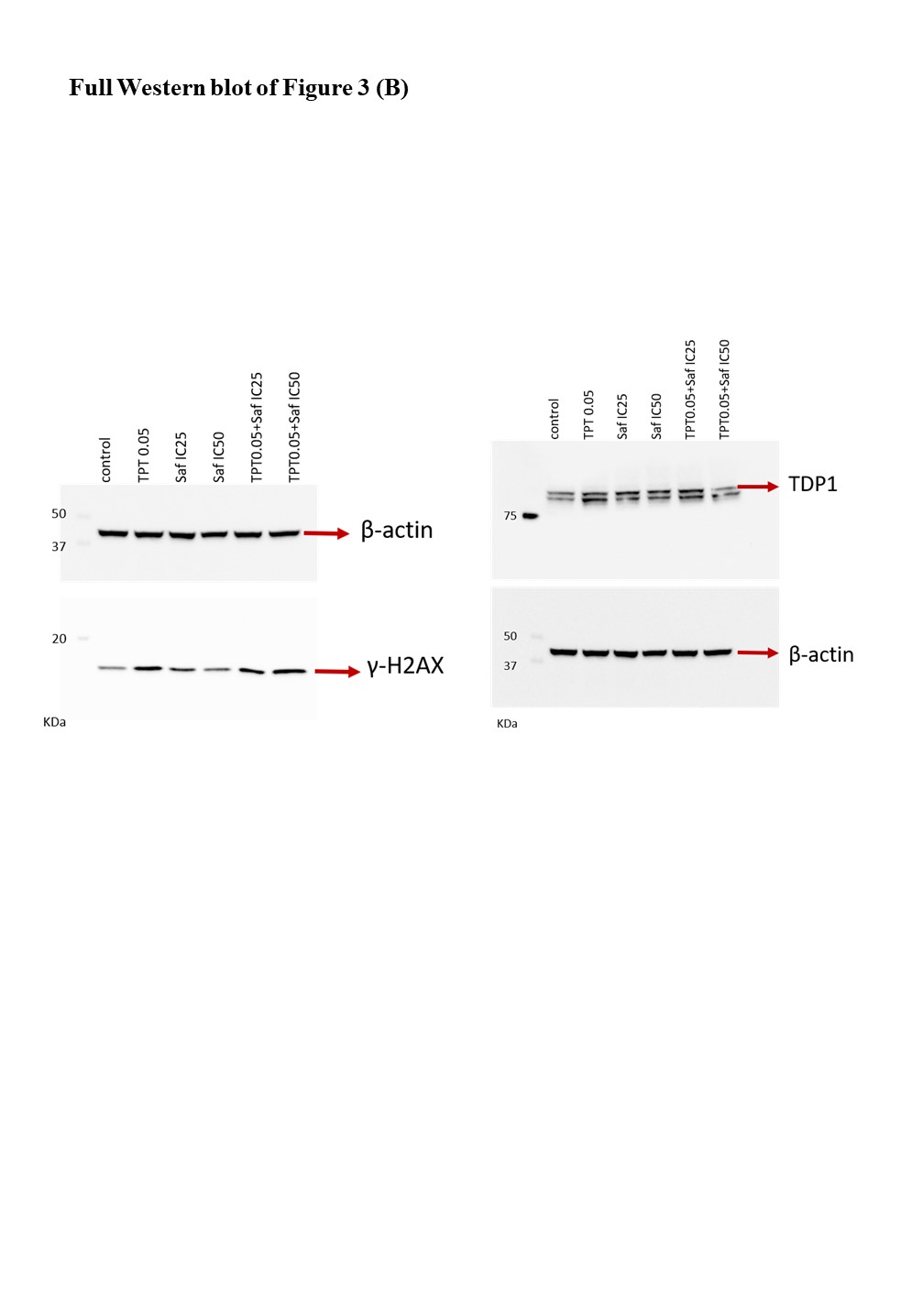

Supplement: Supplementary file 5 [file Image5.JPEG]
